# Poor Performance of the* Chlamydia* Rapid Test Device for the Detection of Asymptomatic Infections in South African Men: A Pilot Study

**DOI:** 10.1155/2016/8695146

**Published:** 2016-04-19

**Authors:** N. S. Abbai-Shaik, T. Reddy, S. Govender, G. Ramjee

**Affiliations:** ^1^HIV Prevention Research Unit, Medical Research Council, 123 Jan Hofmeyer Road, Westville, Durban 3630, South Africa; ^2^Biostatistics Unit, Medical Research Council, 491 Ridge Road, Durban 3630, South Africa; ^3^Department of Epidemiology and Population Health, London School of Hygiene & Tropical Medicine, London WC1E 7HT, UK

## Abstract

*Background*. To the best of our knowledge, there have been no published reports on the diagnostic performance of the* Chlamydia* Rapid Test (CRT) Device for male urine samples. We evaluated the performance of the CRT Device when compared with that of the BD ProbeTec ET PCR Assay in a population of asymptomatic men.* Methods*. The study enrolled 100 men between June and July 2015. From each consenting male, 20–30 mL of urine was collected. Sensitivity and specificity of the rapid test compared to PCR were calculated. All analysis was performed in STATA version 13.* Results*. All men had valid rapid and PCR test results. The test showed a low sensitivity against PCR (20%) (95% CI 3.7–6.2%); however, an excellent specificity was observed (100%) (one sided 97.5% CI: 96.0–100).* Conclusions*. This test was not found to be suitable as a screening tool for genital* Chlamydia* infections in men. Our findings emphasize the need for more sensitive POC tests to be developed since the current approach for the management of STIs in Africa is confounded by poor sensitivity and specificity resulting in many infected individuals not being treated.

## 1. Introduction

Urogenital infection caused by* Chlamydia trachomatis*, the most common bacterial sexually transmitted infection (STI) in the world [1], is associated with nongonococcal urethritis and epididymitis in men [2]. The prevalence of* C. trachomatis* in men in the African region is 2.1% with a reported incidence of 20.9 per a population of 1000 [3]. Additionally,* C. trachomatis* infections have also been shown to increase the risk of Human Immunodeficiency Virus (HIV) acquisition [4]. The documented increase in the number of reported cases of* C. trachomatis* infections has resulted in concerns regarding the effectiveness of current screening programs in parts of the United States as well as other countries [2]. Although nucleic acid amplification tests (NAATs) are significantly more sensitive than enzyme immunoassay based tests, performed with noninvasively collected specimens (urine) and used in screening asymptomatic individuals who represent the bulk of prevalent infections, there are still limitations regarding these tests. The foremost deterrent to the acceptance of NAATs has been their perceived cost [5]. Rapid point of care (POC) tests can be a cost effective strategy for increasing the impact of STI screening interventions. Their greatest advantage is that they can yield results at the patient's first visit, not requiring patient follow-up [6]. Currently, the available rapid tests for the detection of* C. trachomatis* have reasonable specificities [7, 8]. Various types of rapid tests for detecting* C. trachomatis* have been developed. The* Chlamydia* Rapid Test Device (Abon Biopharm (Hangzhou) Co., Ltd.) is a rapid chromatographic immunoassay for the qualitative detection of* C. trachomatis* in male urine samples. At present, the only information available on the test is product literature found on the manufacturer's package insert. To the best of our knowledge, there have been no published reports on the diagnostic performance of this POC test. The objective of this study is therefore to evaluate the diagnostic performance of the* Chlamydia* Rapid Test Device when compared with that of the BD ProbeTec ET PCR Assay (Becton Dickinson Microbiology Systems, USA) for the detection of genital* C. trachomatis* infections in asymptomatic men.

## 2. Methods

### 2.1. Study Population

Men over the age of 18 were invited to participate in this pilot study between June and July 2015. The study population was recruited from the general population in Durban, KwaZulu-Natal, by staff from the South African Medical Research Council's (SAMRC) HIV Prevention Research Unit (HPRU). A total of 100 STI asymptomatic men who are not experiencing discharge from the penis, pain during coitus, pain during urination, genital itching, and testicular pain were enrolled in this study. Men who were allergic to any medication were ineligible since the study included a treatment phase. Men were requested to attend their clinic visit at the HPRU research facility in Westville, Durban. After consenting to participate in this study, the participants were interviewed to collect demographic and clinical data. The ethics committee of the Department of Biomedical and Clinical Technology, Durban University of Technology, as well as the South Medical Research Council (EC002-2/2015) approved the study.

### 2.2. Laboratory Procedures

From each eligible participant, 20–30 mL of urine was collected and transported under the appropriate temperatures to the study laboratory for processing and testing. The urine sample was divided into two proportions. On half was used for the rapid testing and the other half for the BD ProbeTec ET PCR Assay for* Chlamydia* only. The rapid test was performed on the same day as sample collection whereas the PCR was performed in batches within 5–7 days of sample collection. Samples for the PCR were stored at 4°C until processing. The methodology of each of the tests is described below.

### 2.3. *Chlamydia *Rapid Test Device

The urine samples were tested in accordance with the manufacturer's instructions. The* C. trachomatis* antigen was extracted from the sample by addition of the sample extraction buffer to the centrifuge-generated urine pellets and vigorous mixing to obtain a homogenous solution. A second diluent was then added to the homogenous solution and mixed by gentle vortexing. The extracted antigen was then added to the sample window containing the* Chlamydia* coated antibodies. The results were read after 10 minutes. The tests were interpreted qualitatively as per the manufacturers' instructions.

### 2.4. Reference Test

 For Strand-displacement amplification (SDA), BD ProbeTec ET Assay was performed on all samples according to the manufacturer's instructions. The processed sample was added to the Priming Microwell which contained the amplification primers and other reagents necessary for amplification. After incubation, the reaction mixture was transferred to the Amplification Microwell, which contained two enzymes (a DNA polymerase and a restriction endonuclease) necessary for SDA. Results were reported through an algorithm as positive, negative, indeterminate, or equivocal.

### 2.5. Data Analysis

All analysis was performed in STATA version 13. Sensitivity, specificity, positive predictive value (PPV), and negative predictive value (NPV) of the rapid compared to each reference test were calculated. Two sided 95% confidence intervals were computed for all proportions, unless otherwise stated.

## 3. Results and Discussion

The median age of the men was 31 (IQR 23–38) years. All men had valid rapid and PCR test results. There were no invalid rapid tests. Four men had taken antibiotics in the past two weeks. However, these antibiotics did not affect the functioning of the rapid test, since no discordant results were observed between the rapid test and PCR in men who were medicated.

We observed a 10% (95% CI 5.4–17.8) prevalence of asymptomatic genital* chlamydial* infections in this small study population. Reports on the prevalence of asymptomatic* chlamydial* genital tract infections in men have been estimated to range from 3% to approximately 50% [2, 9]. Currently, in South Africa there is limited data on asymptomatic* Chlamydia* infections in men. Despite our small sample size, the findings of our study therefore add to the growing body of knowledge on STI prevalence rates in a male population.

In our study, all 90 individuals who were negative on PCR also tested negative on the rapid test, resulting in a specificity of 100% (one sided 97.5% CI: 96.0–100). Eight out of the 10 PCR positives were incorrectly diagnosed as negative on the rapid test, resulting in a sensitivity of 20% (95% CI 3.7–6.2%) (Table 1). The PPV and NPV were 100% and 91.8%, respectively. To date, available rapid tests for the detection of* C. trachomatis* have shown reasonable specificities but poor sensitivities when compared to other methods [8]. The findings of our study are consistent with a previous published report conducted in a clinical setting with specimens from asymptomatic men [10]. Of the men that tested positive, 60% had returned for their treatment visit which was scheduled within 7 days of their initial clinic visit.

One of the reasons for the observed poor sensitivity could be inadequate microbial load since some of the rapid tests require a high organism load [11]. A distinct relationship between organism load and rapid test performance has been reported by Nadala et al. [12] and Hurly et al. [10]. However, this is not practical in a clinical setting since the microbial load varies for each patient [11] and the detection of the organism is affected by patient factors such as being asymptomatic [6].

## 4. Conclusion

Whilst the sensitivity of the test was low, the specificity of the rapid test was excellent when compared to the reference test and this is in keeping with a previous finding that reported promising levels of specificity for* Chlamydia* Rapid Tests for detecting infections in asymptomatic men [10]. Our findings emphasize the need for more sensitive POC tests to be developed since the current approach for the management of STIs in Africa is confounded by poor sensitivity (30–80%) and specificity (40–80%), resulting in many infected individuals not being treated [13].

## Figures and Tables

**Table 1:** Overall diagnostic performance of the *Chlamydia* Rapid Test Device when compared to the BD ProbeTec ET SDA assay.

	Negative	Positive	Total	Sensitivity % (95% CI)	Specificity % (95% CI)	PPV % (95% CI)	NPV % (95% CI)
*Chlamydia *Rapid Test Device				20% (3.7–62.2)	100% (96.0–100)^*∗*^	100% (15.8–100)^*∗*^	91.8 (84–95.9)
Negative	90	8	98				
Positive	0	2	2				
*Total*	*90*	*10*					

^*∗*^One sided 97.5% confidence interval.
